# Higher miscarriage rate in subfertile women with endometriosis receiving unbiopsied frozen-warmed single blastocyst transfers

**DOI:** 10.3389/fcell.2023.1092994

**Published:** 2023-04-14

**Authors:** M. K. Sachs, S. Makieva, I. Dedes, D. R. Kalaitzopoulos, S. El-Hadad, M. Xie, A. Velasco, R. Stiller, B. Leeners

**Affiliations:** ^1^ Department of Reproductive Endocrinology, University Hospital Zurich, Zurich, Switzerland; ^2^ Department of Gynaecology, University Hospital Zurich, Zurich, Switzerland

**Keywords:** live birth rate, miscarriage rate, endometriosis, pregnancy rate, blastocyst transfer, frozen-warmed, art, IVF/ICSI

## Abstract

**Background:** Assisted reproductive technology treatment is recommended to overcome endometriosis-associated infertility but current evidence is controversial. Endometriosis is associated with lower antral follicle count (AFC) and oocyte yield but similar clinical outcomes compared to controls. Unaffected ovarian stimulation response and embryological outcomes but lower clinical pregnancy and live birth rates and higher miscarriage rates have been reported, implying direct impact on endometrial receptivity. With evidence emerging on the benefit of frozen-warmed and blastocyst stage transfer, we investigated ART outcomes in endometriosis using homogeneous case-control groups.

**Methods:** This is a retrospective observational case-control study including *n* = 66 frozen-warmed unbiopsied single blastocyst transfers of patients with endometriosis and *n* = 96 of women exhibiting idiopathic sterility. All frozen-warmed transfers followed artificial endometrial preparation.

**Results:** In control women, the mean number of oocytes recovered at oocyte pick up was higher compared to women with endometriosis (15.3 ± 7.1 vs. 12.7 ± 5.2, *p* = 0.025) but oocyte maturation index (mature oocytes/total oocytes at oocyte pick up) was significantly higher for endometriosis (48.2% vs. 34.0%, *p* = 0.005). The same was shown for the subgroup of 44 endometriosis patients after endometrioma surgery when compared with controls (49.1% vs. 34.0%, *p* = 0.014). Clinical pregnancy rate was not higher in endometriosis but was close to significance (47.0% vs. 32.3%, *p* = 0.059) while live birth rate was comparable (27.3% vs. 32.3%, *p* = 0.746). Miscarriage rate was higher in the endometriosis group (19.7% vs. 7.3%, *p* = 0.018). A significantly higher AFC was observed in the control group in comparison with the endometriosis group (16.3 ± 7.6 vs. 13.4 ± 7.0, *p* = 0.014). Live birth rate did not differ when comparing all endometriosis cases (*p* = 0.746), ASRM Stage I/II and Stage III/IV (*p* = 0.348 and *p* = 0.888) with the control group but the overall pregnancy rate was higher in ASRM Stage I/II (*p* = 0.034) and miscarriage rate was higher in ASRM Stage III/IV (*p* = 0.030) *versus* control.

**Conclusion:** Blastocyst transfers in women with endometriosis originate from cycles with lower AFC but higher share of mature oocytes than in control women, suggesting that endometriosis might impair ovarian reserve but not stimulation response. A higher miscarriage rate, independent of blastocyst quality may be attributed to an impact of endometriosis on the endometrium beyond the timing of implantation.

## Introduction

Endometriosis is a chronic inflammatory disease characterized by the presence of endometrium-like tissue outside the uterus (Ngô et al., 2009; de Ziegler et al., 2010). It is categorized by three phenotypes: superficial peritoneal lesions, ovarian endometriomas and deep infiltrating endometriosis (Chapron et al., 2019). Disease prevalence (5%–10%) varies as much as its clinical symptoms, ranging from pelvic pain over infertility and fatigue to asymptomatic cases (Ramin-Wright et al., 2018; Taylor et al., 2021; Leuenberger et al., 2022). Among women with infertility the prevalence of endometriosis is estimated to account for 35%–50% (Giudice and Kao, 2004; Prescott et al., 2016; Sanchez et al., 2020). Thus the strong clinical association of infertility and endometriosis has been shown repetitively, the exact link between both remains unclear (Gupta et al., 2008; Tomassetti and D’Hooghe, 2018). In a presumably multifactorial process possible mechanisms reducing fertility in endometriosis are, *e.g.*, the overproduction of embryotoxic cytokines (Akoum et al., 2008), changes of the endometrium itself (de Ziegler et al., 2010), potential immobilization of the sperm by endometrial fluid (Aeby et al., 1996; Pillai et al., 1998) and influence on the ovaries and reproductive organs through space-occupying and cicatrizing effects of endometriomas and other endometriotic lesions (Kasapoglu et al., 2018), surgery-related loss of ovarian tissue and local reactions diminishing the ovarian reserve (de Ziegler et al., 2010; Somigliana et al., 2012). Also oocyte quality may be affected in endometriosis since oocyte morphology and mitochondrial activity has been shown to be impacted (Borges et al., 2015; Xu et al., 2015).

Assisted Reproduction Technology (ART) can be effective in overcoming endometriosis-related infertility, but current evidence is controversial. Unaffected ovarian stimulation response and embryological outcomes as well as similar live birth rates (LBR) in endometriosis patients have been reported but lower clinical pregnancy and higher miscarriage rates, implying also a potential impact on endometrial receptivity, have also been described (Hamdan et al., 2015; Kohl Schwartz et al., 2017; Pallacks et al., 2017; Sanchez et al., 2020; Bishop et al., 2021; Blank et al., 2021).

There is emerging evidence that controlled ovarian hyperstimulation (COH) may carry the risk of a premature window of implantation (WOI) due to alterations of the endometrial receptivity for which elective deferred frozen-warmed embryo transfer may be a suitable solution (24). Further, blastocyst stage transfer in comparison to day 2/3 transfers has become common practice since it carries potential advantages such as lower numbers of embryo transfers while superiority of the cumulative LBR has not clearly been shown yet (De Vos et al., 2016; Glujovsky et al., 2016; Cameron et al., 2020).

Considering the current state of research, we aimed at the investigation of ART outcomes of single blastocyst transfer after frozen-warming in endometriosis patients by selecting methodologically homogeneous case-control groups.

## Materials and methods

In this retrospective observational case-control study 162 patients receiving single frozen-warmed blastocyst transfer at the University Hospital Zurich in Switzerland were included. Of the 162 patients, 66 patients had surgically confirmed endometriosis (20 patients with ASRM (American Society for Reproductive Medicine) stage I/II and 46 patients with ASRM stage III/IV) and 96 exhibited idiopathic sterility. We chose ASRM classification since it was the most commonly used classification in the available surgery reports. The enrollment period comprised all eligible cases between January 2017 and December 2021. We excluded patients of PGT (preimplantation genetic testing)-cycles and those who did not consent to the use of their data. The blastocyst quality was scored according to the Gardner and Schoolcraft classification system (Gardner and Schoolcraft, 1999).

Subjects have given their written informed consent and the protocol has been approved by the “*Kantonale Ethikkommission*” (cantonal ethics review board of Zurich), BASEC Nr. 2018-00795.

### Study and control group

Women with a surgically confirmed diagnosis of endometriosis and subfertility were included in the case group. We chose women with idiopathic infertility to avoid any known pathology associated with local inflammation as eventually present in tubal factor infertility (Karande et al., 1995; Qi et al., 2019; Hill et al., 2020; Mayrhofer et al., 2022) or any endocrinological disease such as PCOS (polycystic ovary syndrome), which might have an impact on oocyte quality. As fertilization rates, which are also related to sperm quality and fertilization method (Opøien et al., 2012) were equal in women with endometriosis and controls we left these women in our analysis (Table 2).

### Controlled ovarian stimulation

Before stimulation, patients received a gestagen (10 mg/d) for 12 days up to 4 weeks in the short or antagonist protocols and a GnRH (gonadotropin-releasing hormone)-agonist (triptorelin, 0.1 mg/d) on cycle day 21 in patients undergoing long protocol COS (controlled ovarian stimulation). For ovarian stimulation either short or long GnRH-agonist protocol or GnRH-antagonist protocol were used according to the appropriate recommendations with either application of hMG (human menopausal gonadotrophins) or recombinant FSH (follicle stimulating hormone (Siristatidis et al., 2015; Lambalk et al., 2017; ESHRE Guideline Group on Ovarian Stimulation Bosch et al., 2020). If at least three follicles with a diameter of ≥17 mm were observed during vaginal ultrasound final oocyte maturation was induced with either 6500 IU (international unit) hCG (Ovitrelle, Merck, Switzerland) or a GnRH-agonist eventually accompanied by about 1600 IU hCG within the GnRH-antagonist protocol. Ultrasound guided oocyte retrieval was performed 36 h after administration of the hCG/GnRH-antagonist trigger.

### Fertilization, embryo culture, vitrification and warming

Depending on the indication, conventional IVF (*in vitro* fertilization)/ICSI (intracytoplasmic sperm injection) was performed five-seven hours following oocyte pick up (OPU). For ICSI, oocytes were first denuded 2 hours following OPU and MII (metaphase II) oocytes were incubated under standard culture conditions (37°C, 6% CO_2_) before insemination. For IVF, oocytes were distributed on a 4-well culture dish, containing one-two oocytes per well, and were inseminated with about 100.000 motile spermatozoa per oocyte. Sixteen-18 hours after IVF or ICSI, oocytes were examined for fertilization and fertilized oocytes with 2 PN were either vitrified at the zygote stage or transferred to appropriate medium under oil and cultured (37°C, 6% CO_2_, 5% O_2_) until being vitrified at the blastocyst stage five-six days after OPU. The vitrification and warming of the zygotes and blastocysts were performed according to the *Cryotop* method using the *Kitazato kit* (KITAZATO CORPORATION, Japan). Warmed zygotes were cultured to the blastocyst stage and either transferred or re-frozen as surplus blastocysts for further transfer cycles. The grading of the blastocyst was performed before vitrification and after warming according to the Istanbul Consensus (ALPHA Scientists In Reproductive Medicine; ESHRE Special Interest Group Embryology) defining 116 ± 2 h post insemination as day 5 of development. No blastocyst was cultured after day 6 of development. Based on the Gardner scoring method the definition of blastocyst quality was as follows; top quality: AA, medium quality: AB, BA, BB and low quality: BC and CB. The expansion of the blastocyst was represented as per Gardner scoring system with 6 being a hatched blastocyst, 5 a hatching blastocyst, 4 a fully expanded blastocyst and 3, 2 or 1 representing medium and low level of expansion respectively (Gardner and Schoolcraft, 1999). All warmed blastocysts were left to recover at least 2 hours prior to embryo transfer.

### Frozen-warmed embryo transfer strategy

In this study we enrolled women with artificial cycles preceding blastocyst transfer. Endometrium preparation was achieved by the application of oral estrogen 6 mg/d followed by the addition of vaginal progesterone 1000 mg/d 5 days prior to blastocyst transfer. Depending on their developmental stage blastocysts were either warmed the day before or on the day of transfer and a single blastocyst was transferred. A quantitative hCG (human chorionic gonadotropin) pregnancy blood test was performed earliest 11 days after the transfer. In case of a positive hCG pregnancy blood test the first pregnancy ultrasound to confirm intrauterine gravidity was scheduled in week six of gestational age.

### Statistical analysis

Main outcome measure was live birth after at least 24 weeks of gestational age. There was no loss to follow up. Secondary outcome measures were overall pregnancy rate (all women with a positive hCG blood test), biochemical pregnancy rate (defined as a positive hCG test but no ongoing clinical pregnancy that was sonographically confirmed), clinical pregnancy rate (defined as a positive hCG blood test and a sonographically confirmed yolk sac and/or embryo), miscarriage rate (defined as pregnancy loss before 20 weeks of gestational age), and oocyte maturation index (Mi = mature (MII) oocytes/total oocytes at OPU).

We analyzed demographic and clinical variables using standard statistical tests: nominal variables were compared by Chi-Square test, serial variables were tested for normal distribution using graphical measures and Kolmogorov-Smirnov/Shapiro-Wilk test followed by independent *t*-test or chi-square test as appropriate. A multinominal regression model was performed to adjust the main and secondary outcome measures for differences in baseline characteristics, whenever needed. Stratification was conducted based on surgically diagnosed endometriosis. Data were presented as mean (±SD) or n (%). A *p*-value < 0.05 was accepted as significant. The statistical analyses and data processing was conducted with SPSS (version 24, IBM, USA).

## Results

### Study population and ovarian stimulation characteristics

Patients’ baseline and treatment cycle characteristics are given in Table 1. Women in the endometriosis group were younger than control women (35.0 ± 4.0 vs. 37.6 ± 4.2, *p* < 0.001). AFC was higher in the control group (16.3 ± 7.6 *versus* 13.4 ± 7.0, *p* = 0.014) (see Table 1). A total of 44 (66%) women diagnosed with endometriosis presented endometrioma. Of all endometriosis patients 20 (30%) were categorized ASRM I-II and 46 (70%) ASRM III-IV. In 36 women (55%) with endometriosis oocytes were fertilized through ICSI which was significantly more than in the control group (32 (33.3%, *p* = 0.006) (Table 2). Other baseline and treatment cycle characteristics were distributed equally between groups.

**TABLE 1:** Patient and treatment cycle characteristics.

	Endometriosis (*n* = 66)	Control (*n* = 96)	*p*-value
		*Idiopathic sterility*	
Age (*years*)	35.0 (±4.0)	37.6 (±4.2)	**<0.001**
Age ≥37 (*years*)	22 (33.30%)	59 (61.50%)	**<0.001**
Age at embryo transfer (*years*)	35.9 (±4.1)	38.7 (±4.0)	**<0.001**
BMI (*kg/m* ^*2*^)	22.3 (±4.3)	23.2 (±4.1)	0.101
AFC	13.4 (±7.0)	16.3 (±7.6)	**0.014**
AMH (*pmol/L*)	14.2 (±9.4)	18.7 (±12.0)	0.073
FSH (*U/L*)	7.8 (±2.8)	7.6 (±2.4)	0.824
TSH (*mU/L*)	1.6 (±0.6)	1.3 (±0.7)	0.787
Endometrial thickness before transfer (*mm*)	8.5 (±1.7)	8.5 (±1.8)	0.641
Endometrioma(s)	44 (66%)	-	-
ASRM I/II	20 (30%)		
ASRM III/IV	46 (70%)		
Stimulation duration (*days*)	12 (±0)	10.0 (±1.4)	0.217
Stimulation dosage (*IU, GnRH agonist or GnRH antagonist*)	2387.5 (±512.7)	2218.8 (±635.4)	0.64
Stimulation protocol			0.080
*Short*	24 (36.40%)	51 (53.10%)	
*Long*	23 (34.80%)	14 (14.60%)	
*Antagonist*	19 (28.80%)	31 (32.30%)	

BMI, body mass index.

AFC, antral follicular count.

AMH, Anti-Mullerian hormone.

FSH, follicle stimulating hormone.

TSH, thyorid stimulation hormone.

ASRM, american society for reproductive medicine.

IU, international units.

GnRH, gonadotropin releasing hormone.

Data presented as n (%) or mean (±SD) using chi-square and independent t-test.

SD, standard deviation.

The bold values indicates the statistical significance.

**TABLE 2:** Treatment cycle primary and secondary outcomes.

	Endometriosis (*n* = 66)	Control (*n* = 96)	*p*-value
		*Idiopathic sterility*	
COC recovered per stimulation cycle	12.7 (±5.2)	15.3 (±7.1)	**0.025** **^a^**
MII oocytes per stimulation cycle	6.2 (±5.7)	5.0 (±4.9)	0.223
Oocyte maturation index (Mi)	48.2%	34.0%	**0.005** **^a^**
Fertilization Rate (per stimulation cycle)	74.7 (±17.0)	75.0 (±15.0)	0.863
Fertilization Method of transferred blastocyst			**0.006**
*IVF*	30 (45.5%)	64 (66.7%)	
*ICSI*	36 (54.5%)	32 (33.3%)	
Embryo Quality (transferred)			0.864
*Top*	37.9%	37.5%	
*Medium*	30.3%	27.1%	
*Low*	31.8%	35.4%	
Embryo expansion grade	4.2 (±0.5)	4.1 (±0.6)	0.565
Pregnancy			**0.015** **^a^**
*Yes*	45 (68.2%)	47 (49.0%)	
*No*	21 (31.8%)	49 (51.0%)	
Biochemical pregnancy (n, %)	14 (21.2%)	17 (17.7%)	0.560
Clinical pregnancy (n, %)	31 (47.0%)	31 (32.3%)	0.059
Miscarriage (*n*, %)	13 (19.7%)	7 (7.3%)	**0.018** **^a^**
Live birth (*n*, %)	18 (27.3%)	31 (32.3%)	0.746
Mode of Delivery			0.151
*Spontaneous Delivery*	2	8	
*Caesarean Section*	14	22	
*Vacuum Extraction*	2	1	
Birthweight (grams)	3458 (±534)	3410 (±536)	0.079

*COC*, cumulus *oocyte complex*. Expression as % of oocytes retrieved within a stimulation cycle (±SD).

*MII,* oocytes = mature oocytes in meiosis II, that were used for IVF/ICSI, treatment.

*Oocyte* maturation *index* (*Mi*) = MII, oocytes/total oocytes at oocyte retrieval from individual stimulation cycle.

Pregnancy: including women with an adequately positive beta-hCG, test 10 days after BC, transfer.

Biochemical pregnancy: including women with only adequately positive beta-hCG, test 10 days after BC, transfer but no clinical pregnancy.

Clinical pregnancy: including women with an adequately positive beta-hCG, test as well as sonographically proven intrauterine pregnancy (yolk sac and/or embryo).

Miscarriage: pregnancy loss before week 20 of gestation age.

Data presented as n (%) or mean (±SD) using chi-square and independent *t*-test.

SD, standard deviation.

The bold values indicates the statistical significance.

^a^ Statistical significance remained after controlling for age on multinominal regression analysis

### Clinical outcomes of frozen-warmed blastocyst transfer

The primary outcome of live birth did not differ between women with endometriosis and those of the control group (see Table 2). Women with endometriosis exhibited a LBR of 27.3% which was not different from women with idiopathic infertility (32.3%, *p* = 0.746), Table 2). Additionally, when comparing stages ASRM I/II and ASRM III/IV with the control group we did not find different LBRs (Table 3). The same was true for the subgroup of women who had undergone laparoscopic surgery with endometrioma removal localized on one or both ovaries, compared to our control population (see Table 4).

**TABLE 3:** Treatment cycle primary and secondary outcomes in subgroup analysis of ASRM stages.

	ASRM mild (I&II) (*n* = 20)	*p*-value (vs. Control)	ASRM severe (III&IV) (*n* = 46)	*p*-value (vs. Control)	Control (*n* = 96)
					*Idiopathic sterility*
COC recovered per stimulation cycle	13.8 (±6.4)	0.806	12.2 (±4.5)	0.300	15.3 (±7.1)
MII oocytes per stimulation cycle	6.2 (±5.7)	0.502	6.2 (±5.3)	0.315	5.0 (±4.9)
Oocyte maturation index (Mi)	43.9% (±35.0)	0.189	50.1% (±35.8)	0.173	34.00%
Fertilization Rate (per stimulation cycle)	67.5 (±15.0)	**0.02**	77.7 (±16.9)	0.408	75.0 (±15.0)
Embryo Quality (transferred)		0.521	35%	0.462	37.5%
*Top*	45%				
*Medium*	15%		37%		27.1%
*Low*	40%		28%		35.4%
Embryo expansion grade	4.2 (±0.5)	0.462	4.3 (±0.5)	0.838	4.1 (±0.6)
Pregnancy		**0.034**	30 (65.2%)	0.408	47 (49%)
*Yes*	15 (75%)				
*No*	5 (25%)		16 (34.8%)		49 (51%)
Biochemical pregnancy	4 (20.0%)	0.809	10 (21.7%)	0.567	17 (17.7%)
Clinical pregnancy	11 (55.0%)	0.055	20 (43.5%)	0.193	31 (32.3%)
Miscarriage	4 (20%)	0.078	9 (19.6%)	**0.030**	7 (7.3%)
Live birth	7 (35%)	0.348	11 (23.9%)	0.888	31 (32.3%)
Mode of Delivery		0.122	1	0.348	8
*Spontaneous Delivery*	0				
*Caesarean Section*	6		9		22
*Vacuum Extraction*	1		1		1
Birthweight (grams)	3709 (±354)	**0.035**	3283 (±583)	0.703	3410 (±536)

COC, cumulus oocyte complex. Expression as % of oocytes retrieved within a stimulation cycle (±SD).

MII, oocytes = mature oocytes in meiosis II, that were used for IVF/ICSI, treatment.

Oocyte maturation index (Mi) = MII, oocytes/total oocytes at oocyte retrieval from individual stimulation cycle.

Pregnancy, including women with an adequately positive beta-hCG, test 10 days after BC, transfer.

Biochemical pregnancy, including women with only adequately positive beta-hCG, test 10 days after BC, transfer but no clinical pregnancy.

Clinical pregnancy, including women with an adequately positive beta-hCG, test as well as sonographically proven intrauterine pregnancy (yolk sac and/or embryo).

Miscarriage, pregnancy loss before week 20 of gestation age.

Data presented as *n* (%) or mean (±SD) using chi-square and independent t-test.

SD, standard deviation.

The bold values indicates the statistical significance.

**TABLE 4:** Treatment cycle primary and secondary outcomes in subgroup analysis of endometriosis patients after endometrioma surgery.

	Endometrioma (s) (*n* = 44)	Control (*n* = 96)	*p*-value
		*Idiopathic sterility*	
AFC	14 (±7.0)	16.3 (±7.6)	0.121
Embryo Quality (transferred)			0.195
*Top*	38.60%	37.50%	
*Medium*	38.60%	27.10%	
*Low*	22.70%	35.40%	
Embryo Expansion (scale 1–6)	4.3 (±0.5)	4.1 (±0.6)	0.627
COC recovered per stimulation cycle	12.5 (±5.0)	15.3 (±7.1)	0.168
MII oocytes per stimulation cycle	6.3 (±5.6)	5.0 (±4.9)	0.454
Oocyte maturation index (Mi)	49.1%	34.0%	0.014*
Fertilization Rate (per stimulation cycle)	74.9 (±16.0)	75.0 (±15.0)	0.737
Clinical pregnancy (n, %)	20 (45.5%)	32 (33.30%)	0.301
Live birth (n, %)	9 (25.7%)	22 (22.9%)	0.897
Miscarriage (n, %)	18.20%	7.30%	0.056

Endometrioma (s), Women underwent laparoscopic surgery before controlled ovarian stimulation by indication of endometrioma (s) localized at one or both ovaries.

AFC, antral follicular count.

COC, cumulus oocyte complex. Expression as % of oocytes retrieved within a stimulation cycle (±SD).

MII, oocytes = mature oocytes in meiosis II, that were used for IVF/ICSI, treatment.

Oocyte maturation index (Mi) = MII, oocytes/total oocytes at oocyte retrieval from individual stimulation cycle.

Pregnancy, including women with an adequately positive beta-hCG, test 10 days after BC, transfer.

Biochemical pregnancy, including women with only adequately positive beta-hCG, test 10 days after BC, transfer but no clinical pregnancy.

Clinical pregnancy, including women with an adequately positive beta-hCG, test as well as sonographically proven intrauterine pregnancy (yolk sac and/or embryo).

Miscarriage, pregnancy loss before week 20 of gestation age.

Data presented as n (%) or mean (±SD) using chi-square and independent t-test.

SD = Standard deviation.

*Statistical significance remained after controlling for age on multinominal regression analysis.

The bold values indicates the statistical significance.

With regards to secondary outcomes, we observed a difference in the overall pregnancy rate, combining biochemical and clinical pregnancies, between endometriosis patients and those with idiopathic infertility, revealing a higher overall pregnancy rate in endometriosis patients (68.2% vs. 49.0%, *p* = 0.015) (Table 2). When analyzed separately, biochemical and clinical pregnancy rates did not differ between the groups (Table 2). Of note, despite no difference in the LBR, women with endometriosis experienced significantly more miscarriages than the controls (19.7% *versus* 7.3%, *p* = 0.018, Table 2). The subgroup analysis showed that women with mild ASRM scores have higher pregnancy rates while women with severe ASRM scores exhibited more miscarriages (Table 3).

Concerning oocyte and embryo quality measures, neither embryo quality nor embryo expansion grade differed between groups. Interestingly, the number of retrieved oocytes per simulation cycle was higher in the control group (12.7 ± 5.2 *versus* 15.3 ± 7.1, *p* = 0.025, Table 2) while the number of mature oocytes did not differ between groups (6.2 ± 5.7 *versus* 5.0 ± 4.9, *p* = 0.223, Table 2) and the Mi was significantly higher in endometriosis patients (48.2% *versus* 34.0%, *p* = 0.005, Table 2). A higher Mi was also shown for the subgroup of women with endometrioma (49.1% *versus* 34.0%, *p* = 0.014, Table 4). Fertilization rate, calculated based on the number of fertilized mature oocyte per stimulation cycle, was neither inferior in the endometriosis group (74.7% ± 17.0 *versus* 75.0% ± 15.0, *p* = 0.863, Table 2) nor in the endometrioma group (74.9% ± 16.0 *versus* 75.0% ± 15.0, *p* = 0.737, Table 4). In the ASRM I/II group fertilization rate was lower than in the control group (67.5% (±15.0) *versus* 75.0% ± 15.0, *p* = 0.02, Table 3).

To sum up, our main results show a similar live birth rate in endometriosis and control women as well as a higher miscarriage rate in women with an endometriosis diagnosis.

### Multinominal logistic regression of oocyte maturation index and pregnancy rate

Women with endometriosis are significantly younger (*p* < 0.001, Table 1) than the control and their Mi is higher (*p* = 0.005, Table 2) as well as their overall pregnancy rate (*p* = 0.015, Table 2). Therefore we performed a multinominal logistic regression analysis, revealing that Mi (Table 2, asterisk), overall pregnancy rate (Table 2, asterisk) as well as miscarriage rate (Table 2, asterisk) remains to be significant higher in endometriosis patients when correcting for the age ≥37 years. Similarly, Mi remained significantly higher in endometrioma patients compared to women with idiopathic infertility Table 4, asterisk). Data was not adjusted for AFC since all cycles were artificial thawing cycles where AFC is not assumed to have a relevant impact on embryo implantation and further outcomes.

## Discussion

Women with endometriosis revealed same LBR in comparison with women diagnosed with idiopathic sterility after frozen-warmed single non-biopsied blastocyst transfers. Furthermore, women with endometriosis showed a higher oocyte maturation index (Mi) and overall pregnancy rate while experiencing a higher share of miscarriages.

Previous studies with a focus on LBR as an outcome measure in endometriosis patients undergoing ART have revealed heterogeneous results. In line with our study results others report a similar LBR in women with endometriosis compared with controls (isolated male factor infertility and non-infertile patients with PGT-SR (structural rearrangements)) undergoing frozen warmed euploid blastocyst transfer (Bishop et al., 2021), as also shown by a study comparing day 2 embryo transfer outcomes in endometriosis compared with non-endometriosis patients (Feichtinger et al., 2019). Further, same overall LBR in endometriosis patients was confirmed in another systematic review and meta-analysis similarly showing no difference in LBR in the total group of endometriosis patients in comparison with different control groups (e.g., unexplained infertility, tubal factor, all other infertility causes) (Hamdan et al., 2015). A recent matched-cohort study, matching women with endometriosis with those without, of similar age, type of stimulation and AMH (anti-mullerian hormone) levels, also reported similar LBR and clinical pregnancy rates (Invernici et al., 2022). Coherent results can be found in a recent study on women with advanced endometriosis that underwent frozen-thawed embryo transfers (day 3 embryos or blastocyst) indicating that LBR in the disease group was not different (Li et al., 2020).

In contrast, subfertile endometriosis patients undergoing ART with fresh transfer of embryos (IVF or ICSI) between days 2 and 5 revealed an up to 24% lower LBR compared to women with unexplained subfertility in a cohort study (Muteshi et al., 2018). Similarly women with endometriosis undergoing fresh embryo transfer on day 5 (IVF/ICSI) compared with sole male factor infertility depicted a lower LBR (Blank et al., 2021).

It is possible that LBR outcomes in endometriosis patients may be altered in particular in advanced endometriosis stages. Declining LBR in women with advanced stages of endometriosis in comparison with mild endometriosis and tubal factor infertility undergoing embryo transfer after IVF/ICSI have been reported (Kuivasaari et al., 2005). In the above mentioned meta-analysis and systematic review on IVF and/or ICSI LBR was not different in the total group of endometriosis patients, but LBR was inferior in the subgroup of severe endometriosis cases (Hamdan et al., 2015).

Combined, these studies point towards similar LBR in subfertile women with endometriosis undergoing ART. Restrictively incoherent patient populations make a direct comparison of studies difficult but also offer a broad picture of the patient population undergoing ART: methods of fertilization vary, control groups differ, day of embryo transfer, stages and diagnostic measures of endometriosis as well as fresh or frozen-warmed transfer, just to name a few. To our best knowledge, the study we present is the first focusing on the common clinical scenario of single, non-biopsied frozen-warmed blastocyst transfer and LBR as primary outcome measure in women with endometriosis.

Our finding of a significantly higher number of oocytes retrieved per stimulation cycle in the control group is in line with findings of previous studies (Hamdan et al., 2015; Muteshi et al., 2018; Feichtinger et al., 2019; Horton et al., 2019). This can be attributed to lower AFC and AMH values in endometriosis patients (Kasapoglu et al., 2018; Li et al., 2020), which is a result from endometriosis (advanced stages and/or endometrioma) itself and from surgery (Tian et al., 2021).

The oocyte maturation index (Mi) may be an indicator for the clinical pregnancy rate and LBR (Behr et al., 2012; Parrella et al., 2019). Surprisingly, the Mi per stimulation cycle of our endometriosis cohort was even higher than in the control, possibly outweighing the overall lower number of retrieved oocytes by an adequate quality, assuming that a high Mi leads towards potent embryos and viable pregnancies. “They are few but they are mature”—maybe not only the maturation process of the oocytes seems to be mainly affected in endometriosis but the potential to produce oocytes during COS? Even our subgroup of women who underwent prior surgical endometrioma removal had a higher Mi than the control. It is possible that the answer lies in the choice of the control group, as a study looking at natural cycle IVF outcomes of women with unexplained sterility compared with tubal factor infertility and peritoneal endometriosis showed adverse outcomes in unexplained sterility, in terms of follicular periovulatory growth, fertilization and pregnancy rates and therefore possibly reduced oocyte quality (Omland et al., 2001). However our study groups equally showed satisfactory fertilization rates when comparing with literature (Lee et al., 2017; Song et al., 2021).

Fertilization rates in women with endometriosis were shown to be reduced in a review and meta-analysis (Horton et al., 2019), while Sanchez et al. report same fertilization rates and embryo quality but reduced ongoing pregnancy rates in these women (Sanchez et al., 2020).

Commonly used oocyte and embryo quality rating tools are insufficiently precise to portray presumptive defects of oocytes and embryos taking place on a molecular level. In our cohort embryo quality rating did not differ between groups but pregnancy rates were higher as well as miscarriage rates in subfertile women with endometriosis. To look further into oocyte and embryo quality should definitively be an aim, as, e.g., impaired mitochondrial structure and function impairments may be seen as potential adverse effects of endometriosis in fertility (Xu et al., 2015). Several studies do assume that oocyte and as a result embryo quality is inferior in subfertile endometriosis patients (Da Broi et al., 2014; Borges et al., 2015; Xu et al., 2015; Da Broi et al., 2018; Da Luz et al., 2022). Oocyte quality can be assessed indirectly as cumulus cells and follicular fluid are closely linked with the oocyte as they contribute to its maturation and development (Dumesic et al., 2015). The transcriptome of cumulus cells has been shown to be altered in endometriosis patients when compared with a control group of tubal abnormalities or male factor infertility, which may be related to lower oocyte quality (Da Luz et al., 2022). Another indirect proof can be found in a study where immature bovine oocytes, that underwent *in vitro* maturation with follicular fluid from infertile women with endometriosis, revealed higher meiotic abnormalities and stayed in metaphase I significantly more often, when compared with immature bovine oocytes cultured in follicular fluid from healthy women (Da Broi et al., 2014). Further studies report affected oocyte morphology (extra-cytoplasmatic defects) (Borges et al., 2015) and impaired mitochondrial structure (Xu et al., 2015) in women with endometriosis.

A recent study on aneuploid rates and euploid frozen blastocyst transfer in women with endometriosis and two control groups (isolated male factor infertility and ART indication based on single-gene disorders) found no difference in pregnancy outcomes, when controlling for embryo quality by using euploid frozen embryo transfer cycles only. Of note, aneuploidy rates were not different in endometriosis patients (Bishop et al., 2021). Since our endometriosis population has a mean age of 35 years and the control group of 37.6 years and no other genetic disease was present, no blastocyst biopsy was performed in our cohort and based on the mentioned study, we conclude that genetic causes of the embryo may not have influenced neither pregnancy, miscarriage nor LBR.

Nowadays it is also common practice in ART clinics to transfer frozen-warmed blastocysts for different reasons: no pregnancy after fresh embryo transfer, ovarian hyperstimulation syndrome and in particular in endometriosis patients embryo transfer may be delayed due to planned endometriosis surgery before embryo transfer if persisting endometriosis symptoms are present and/or endometrioma surgery is inevitable and is potentially threatening the ovarian reserve. To our knowledge, there is a lack of reliable data on pregnancy rates after fresh vs. frozen thawed embryo transfer in endometriosis related infertility but in a retrospective study of women without endometriosis treated with an antagonist protocol live birth rates in frozen-warmed embryo transfers where shown to be higher compared with fresh embryo transfer (Fan et al., 2022), while others did not show a benefit of a freeze-all strategy in comparison with a fresh embryo transfer (Feichtinger et al., 2019; Wong et al., 2021). As frozen-thawed embryo transfer is common practice it was chosen as inclusion criteria in this study, even though further investigation on its benefit is required.

In conjunction with the higher miscarriage rates, not only oocyte and embryo quality but also the role of the endometrium may be a relevant culprit. Higher miscarriage rates in woman with endometriosis have been reported repetitively throughout the literature, and also our results validate this link (Horton et al., 2019). Pallacks et al. report that endometriosis doubles the odds for miscarriage while he unfortunately did not report on LBR (Pallacks et al., 2017). Kohl Schwartz et al. also reported more miscarriages in endometriosis patients while the number increased with ASRM stage (Kohl Schwartz et al., 2017), which coincides with the outcome of this study. However, other studies reported similar miscarriage rates (Hamdan et al., 2015; Leonardi et al., 2016; Bishop et al., 2021). For example, a large matched case-control study of women undergoing IVF with and without endometriosis, including fresh as well as thawing cycles, revealed similar miscarriage rates. (Leonardi et al., 2016). None of the mentioned studies including ours can explain the exact link between miscarriages and endometriosis, but the clinical association between both supports the concept of diminished endometrial receptivity. As endometriosis is known to be a systemic disease an adverse impact of inflammation on the endometrium is suspected and in this context implantation failure. As endometrial receptivity is tested increasingly, the literature does name specific defective pathways in the endometrium of endometriosis patients (Miller et al., 2017). In conjunction with endometriosis as an inflammatory disease, local cytokine expression is upregulated in the endometrium and is known to influence local receptor expression playing a role in embryo attachment (Lessey and Kim, 2017). A recent review by Vigano’ et al. suspects COS to induce a premature window of implantation (WOI) due to higher than normal circulating levels of estrogen and progesterone, which may consequently result in an already closed WOI at the time of fresh blastocyst transfer (Viganò et al., 2020). Our endometriosis cohort shows a higher implantation rate, as reflected by the overall pregnancy rate when compared with the group of unexplained sterility. In particular the ongoing implantation may be altered in endometriosis as the three initial steps of implantation (apposition, adhesion and invasion) (Bischof and Campana, 1997; Kim and Kim, 2017) seem to be successful. The exact mechanisms of miscarriage in endometriosis remains unclear but the association of endometriosis with a highly inflammatory (e.g., increased NK cells and proinflammatory cytokines) and pro-angiogenic (e.g., PIGF) microenvironment has been demonstrated in peritoneal fluid of endometriosis patients (Kolanska et al., 2021) and could impact the pregnancy beyond the first steps of implantation.

In fertility clinics frozen-warmed transfers are common due to prevention of, e.g., overstimulation syndrome or if a fresh embryo transfer did not result in a viable pregnancy. As we only included frozen warmed blastocyst transfers in this study, we assume that we overcame the potential shift of WOI induced by COS in endometriosis patients.

Additionally it is well known that severe endometriosis appears in conjunction with adenomyosis, representing a risk factor for miscarriages and other adverse pregnancy outcomes (Chapron et al., 2017; Younes and Tulandi, 2017; Berlanda et al., 2022). Another possible explanation to account for the increased miscarriage rate in women with endometriosis in the absence of defective receptivity could be an altered uterine microbiome. Indeed, Ortiz et al. (Ortiz et al., 2021), have recently demonstrated differences in the endometrial metabolomics profile while comparing women with and without endometriosis (Ortiz et al., 2021). Hence, it is tempting to develop a novel hypothesis, that a dysfunctional metabolome in the uterine cavity fails to support further development of the implanted blastocyst. This may be especially affect women who suffer from endometriosis. It would be interesting to explore this notion further to explain and manage miscarriages related to endometriosis.

Interestingly, in our study the overall pregnancy rate was still higher in the endometriosis group. We find this outcome challenging to compare with others, as current literature usually only reports clinical pregnancy rates. Authors report on same clinical pregnancy rates and separately same positive hCG-levels after frozen-thawed euploid blastocysts in endometriosis patients (Bishop et al., 2021) and same clinical pregnancy rates in endometriosis patients, compared with unexplained sterility undergoing IVF embryo transfer (Muteshi et al., 2018) opposed to more frequent positive hCG-levels and clinical pregnancies in the control group of male infertility, in comparison with endometriosis (Blank et al., 2021), lower clinical pregnancy rates in a review on ART outcomes and endometriosis (Hamdan et al., 2015), and a lower cumulative pregnancy rate in rASRM stage III/IV endometriosis compared with tubal infertility (Kuivasaari et al., 2005). We speculate that potentially diminished oocyte/embryo quality as well as the altered receptivity of the endometrium and its inflammatory environment, may affect embryonic development beyond the first steps of implantation and can be seen as a reason for the defective long-term embryo development, apparent through satisfactory clinical pregnancy rates at first that do not retain and result in a higher percentage of miscarriage rates in the further process of embryo development. Further research is needed to elucidate interactions between the embryo and the endometrium with underlying endometriosis in more detail.

### Study strengths and limitations

Our study has several strengths and limitations. First, this study cohort is a homogenous case-control group of excellent data quality where only women with endometriosis-related infertility and women with idiopathic sterility receiving single non-biopsied frozen-warmed blastocyst transfer were included. Second, there has not been any loss to follow-up regrading pregnancy rates, miscarriage rates or LBR. Third, all women enrolled underwent a single frozen-warmed blastocyst transfer so that potential bias of embryo developmental stage could be excluded. To our knowledge, this is one of few studies to report on LBR and miscarriage rate in women with endometriosis undergoing frozen warmed, in particular unbiopsied single blastocyst transfer.

We would like to acknowledge the following limitations. The cohort size limits the statistical analysis of our data. Additionally, the study is of retrospective nature. As semen quality influences fertilization rates differences in frequencies of IVF/ICSI in patients and controls might have influenced fertilization rates, but fertilization rates were similar in both groups. Also, currently there is neither evidence that ICSI (Buckett et al., 2008) is associated with higher miscarriage rates as found in our group of women with endometriosis nor with altered LBR (Song et al., 2021). Moreover, the study findings might not apply when cleavage or fresh transfers are concerned. The choice of couples with idiopathic sterility as the control group may be debated, as different underlying currently still unknown pathologies may influence treatment success. This may also affect women who suffer from endometriosis.

## Conclusion

Frozen-warmed single blastocyst transfers in women with endometriosis originate from cycles with lower AFC and number of oocytes at OPU but higher share of mature oocytes than in control women, suggesting that endometriosis might impair ovarian reserve but not stimulation response. A higher miscarriage rate may be attributed to an impact of endometriosis on the blastocyst quality and/or endometrium beyond the timing of implantation. Encouragingly, ART can overcome sterility in woman with endometriosis, as we show no differences in LBR between groups.

## Data Availability

The raw data supporting the conclusion of this article will be made available by the authors, without undue reservation.
